# Immunomodulatory biomaterial-based wound dressings advance the healing of chronic wounds via regulating macrophage behavior

**DOI:** 10.1093/rb/rbac065

**Published:** 2022-09-06

**Authors:** Ana Beatriz Sousa, Artur P Águas, Mário A Barbosa, Judite N Barbosa

**Affiliations:** i3S—Instituto de Inovação e Investigação em Saúde, Universidade do Porto, Rua Alfredo Allen, 208, 4200-125 Porto, Portugal; INEB—Instituto de Engenharia Biomédica, Rua Alfredo Allen, 208, 4200-125 Porto, Portugal; ICBAS—Instituto de Ciências Biomédicas Abel Salazar, Universidade do Porto, Rua de Jorge Viterbo Ferreira, 228, 4050-313 Porto, Portugal; ICBAS—Instituto de Ciências Biomédicas Abel Salazar, Universidade do Porto, Rua de Jorge Viterbo Ferreira, 228, 4050-313 Porto, Portugal; UMIB—Unit for Multidisciplinary Biomedical Research of ICBAS—Instituto de Ciências Biomédicas de Abel Salazar, Universidade do Porto, Rua de Jorge Viterbo Ferreira, 228, 4050-313 Porto, Portugal; i3S—Instituto de Inovação e Investigação em Saúde, Universidade do Porto, Rua Alfredo Allen, 208, 4200-125 Porto, Portugal; INEB—Instituto de Engenharia Biomédica, Rua Alfredo Allen, 208, 4200-125 Porto, Portugal; ICBAS—Instituto de Ciências Biomédicas Abel Salazar, Universidade do Porto, Rua de Jorge Viterbo Ferreira, 228, 4050-313 Porto, Portugal; i3S—Instituto de Inovação e Investigação em Saúde, Universidade do Porto, Rua Alfredo Allen, 208, 4200-125 Porto, Portugal; INEB—Instituto de Engenharia Biomédica, Rua Alfredo Allen, 208, 4200-125 Porto, Portugal; ICBAS—Instituto de Ciências Biomédicas Abel Salazar, Universidade do Porto, Rua de Jorge Viterbo Ferreira, 228, 4050-313 Porto, Portugal

**Keywords:** wound healing, chronic wounds, macrophage, immunomodulatory biomaterials

## Abstract

Successful wound healing is a process that has three overlying phases: inflammatory, proliferative and remodeling. Chronic wounds are characterized by a perpetuated inflammation that inhibits the proliferative and remodeling phases and impairs the wound healing. Macrophages are key modulators of the wound healing process. Initially, they are responsible for the wound cleaning and for the phagocytosis of pathogens and afterwards they lead to the resolution of the inflammatory response and they express growth factors important for angiogenesis and cytokines and growth factors needed for cell proliferation and deposition of extracellular matrix. The phenotype of the macrophage changes gradually throughout the healing process from the initial M1 pro-inflammatory phenotype characteristic of the acute response to the M2 pro-regenerative phenotype that allows an accurate tissue repair. In chronic wounds, M1 pro-inflammatory macrophages persist and impair tissue repair. As such, immunomodulatory biomaterials arise as promising solutions to accelerate the wound healing process. In this review, we discuss the importance of macrophages and their polarization throughout the different phases of wound healing; macrophage dysfunction in chronic wounds and the use of immunomodulatory biomaterials to overcome the critical problem of chronic wounds—the continued inflammatory phase that impairs healing.

## Graphical Abstract

**Figure:**
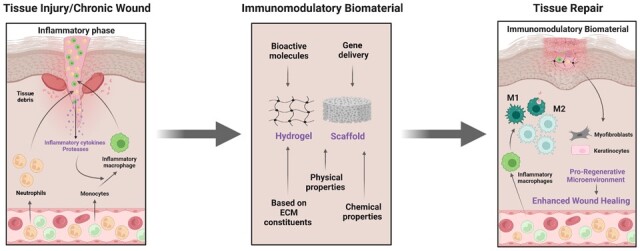

## Chronic wounds and their dimension

When the normal wound healing progress is disrupted, a chronic wound is developed. There is not, however, a clear definition for chronic wound in the literature. It is generally accepted that a wound that fails to advance through the normal sequence of the wound repair phases, within a period around 3 months, the wound is considered to be chronic [1]. Potentially, all wounds can become non-healing chronic wounds. They are different in etiology and can thus be divided into four main categories: arterial, diabetic, pressure and venous ulcers. Although different, chronic wounds present similar features, such as high levels of pro-inflammatory cytokines, profuse neutrophil infiltration with its associated reactive oxygen species (ROS), persistent infections that can lead to the formation of biofilms highly antibiotic-resistant and senescent cells that do not react to reparative stimuli [2–4]. Numerous clinical conditions can delay wound healing, such as diabetes, obesity, ageing, chronic disease or vascular insufficiency. Local factors such as pressure, infection and edema will also affect the healing process [4].

Chronic wounds represent a health problem with overwhelming consequences for patients, and account for major costs to healthcare systems and societies. These disturbing wounds have a huge negative impact on the patient’s quality of life, and can lead to loss of mobility and sleep deprivation, anxiety and depression and contribute to an increased risk of amputation. It is estimated that about 2–4% of the overall healthcare expenses in developed countries are due to treatment costs of chronic wounds. Besides the direct health care costs, indirect costs such as sick leave, productivity losses and early retirement are also substantial [5–7].

Chronic wounds represent a ‘silent epidemic’ amongst the world population and they affect the quality of life of millions globally [4]. In the USA, more than 6 million people are affected by chronic wounds, and these numbers have been rising due to the aging population and to the increased prevalence of diabetes mellitus [8]. In Europe, it is expected that 2% of the total population will be affected by chronic wounds [9]. In the UK, chronic wounds affect 1% of the adult population rising to 5% in the population over 65 years [10].

Usually, chronic wounds are treated with dressings in an attempt to achieve a faster regeneration. Biomaterials and biomaterial-based immunomodulation have emerged as potential solutions to reduce healing time and to improve the patient’s quality of life [10, 11]. This review will discuss recent advances in wound healing through the development of immunomodulatory biomaterials.

## Phases of wound healing

**Figure 1.**
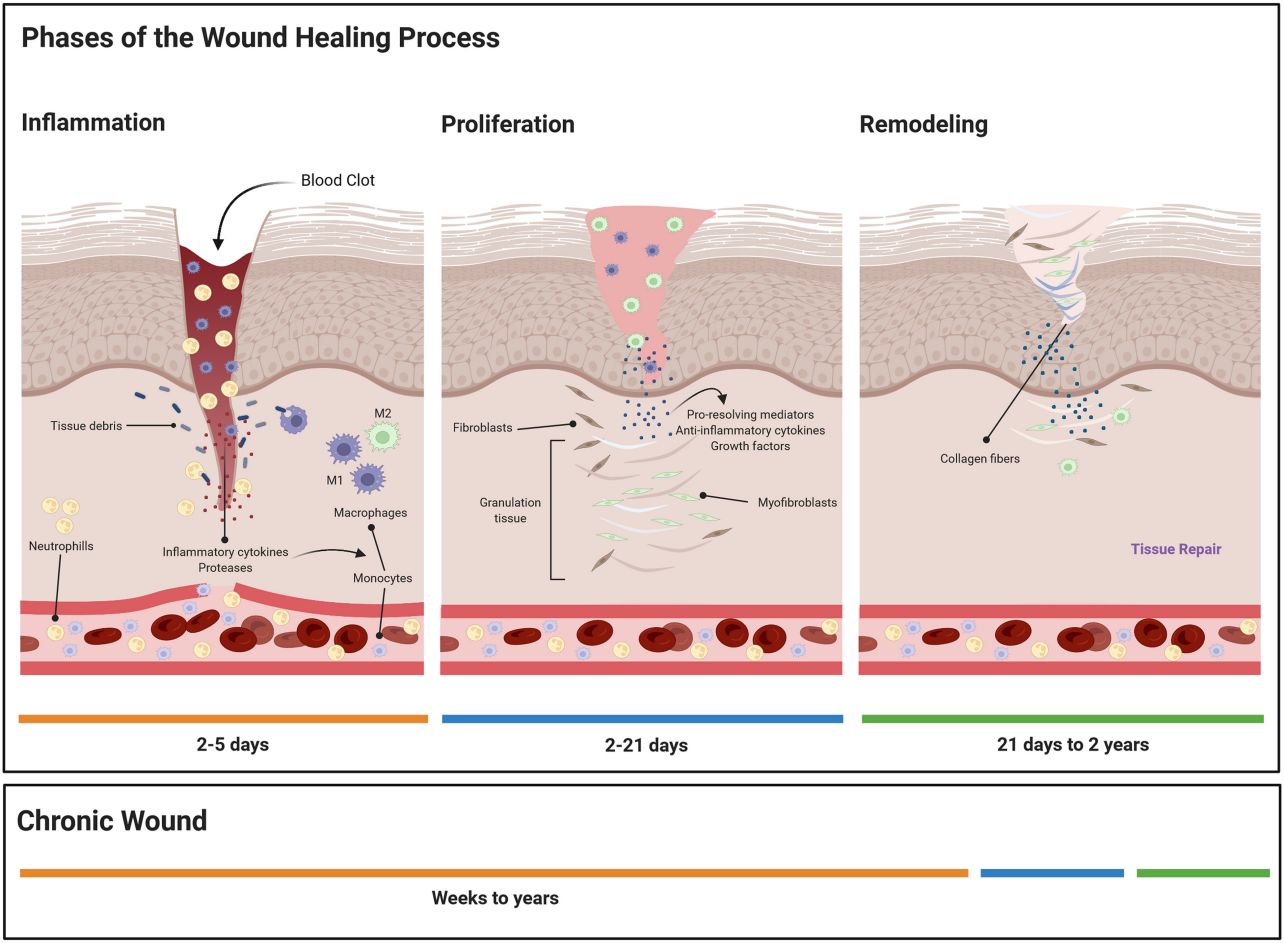
Phases of wound healing. Inflammation is characterized mainly by the formation of a fibrin clot followed by inflammatory cell infiltration, namely PMNs and monocytes that will differentiate in macrophages and by wound debridement. Macrophages will also secrete cytokines and growth factors that will stimulate fibroblasts proliferation. In the proliferation phase, fibroblasts synthetize collagen, there is ECM deposition and angiogenesis occur, and granulation tissue is formed. The last phase is the remodeling in which collagen in the wound matures and strengthens. Chronic wounds are characterized by an abnormal perpetuated inflammatory phase and therefore do not proceed to the subsequent phases of wound healing.

Wound healing involves replacing damaged cellular structures and tissue layers, which is a very dynamic and complex process. Independently of the cause of injury, wound healing progresses in three distinct phases that help our understanding of the biological processes that take place in the wounded tissue and in the surrounding area: (i) inflammatory, (ii) proliferative and (iii) remodeling phases (Fig. 1). Some authors consider four phases with an initial hemostasis phase, but in our view, the hemostasis initial response is part of the inflammatory phase. It is important to understand that to achieve a successful healing of a wound; all phases must follow in the correct sequence and period [12].

### The inflammatory phase

Immediately following injury, hemostasis begins with vascular constriction and the formation of a fibrin clot. The surrounding tissues and the clot will release growth factors and pro-inflammatory cytokines such as fibroblast growth factor (FGF), epidermal growth factor (EGF), platelet-derived growth factor (PDGF) and transforming growth factor (TGF)-β, which will allow the onset of the inflammatory phase [13].

The inflammatory phase starts with the migration of inflammatory cells to the wound area. Injured cells release the so-called ‘danger signals’ such as damage-associated molecular patterns (DAMPs), hydrogen peroxide (H_2_O_2_), lipid mediators and chemokines that will provide signals for the recruitment of inflammatory cells, especially polymorphonuclear leukocytes (PMNs) [14]. There is a progressive infiltration of PMNs, macrophages and lymphocytes. PMNs will clear invading microorganisms and cellular debris of the wounded area. They will also release ROS, and proteases, that can lead to additional injury. PMNs also release different mediators such as tumor necrosis factor (TNF)-α, interleukin (IL)-1 and IL-6, which amplify the inflammatory response and stimulate vascular endothelial growth factor (VEGF) and IL-8 for an adequate repair response [15]. Progressively, PMNs will be replaced by macrophages as monocytes migrate into the wound and differentiate into macrophages [4].

Initially, macrophages release cytokines that will induce the recruitment and activation of additional leukocytes to the wounded area. They are also responsible for the clearance of apoptotic cells such as PMNs [16]. After undergoing a phenotypic switch, macrophages will stimulate fibroblasts, keratinocytes, and angiogenesis creating a microenvironment favorable to the promotion of tissue repair. After approximately 72 h, macrophages will be the predominant cells at the wound site, and will release growth factors such as VEGF and PDGF and cytokines such as IL-1β, IL-6 and TNF-α that promote the activation and migration of other cells such as fibroblasts and endothelial cells to the wound site [15]. Therefore, macrophages have a crucial role in the transition from the inflammatory to the proliferative phase of wound healing [17, 18]. The resolution of the inflammatory phase is an active process regulated by several factors such as the specialized pro-resolving mediators (SPMs) derived from essential fatty acids that provide apoptotic or chemotactic stimuli to inflammatory cells that eventually shifts progress toward the proliferation phase [19].

### The proliferative phase

The proliferative phase takes place approximately from Days 4 to 21 after injury. In this phase, it is important to cover the wound area, the formation of granulation tissue and the restoration of the vascular network. Therefore, deposition of extracellular matrix (ECM), angiogenesis and epithelization occur [14, 20].

Inflammatory cells and platelets release growth factors such as TGF-β and PDGF that will attract fibroblasts and myofibroblasts of the surrounding tissue to the wounded area. In the wound microenvironment, these cells proliferate and produce the matrix proteins such as hyaluronan, proteoglycans, fibronectin and collagen. After some days, extracellular matrix accumulates and further support cell migration, which is very important in the repair process [21].

Fibroblasts undergo a phenotypic change to myofibroblast that will attach firmly to fibronectin and collagen of the extracellular matrix, leading to wound contraction and approximation of the wound edges, an important aspect of the repair process [12]. Fibroblasts will also release growth factors to stimulate other cells, namely, they stimulate epithelialization from keratinocytes through keratinocyte-derived growth factor (KGF) secretion. Additionally, endothelial cells produce basic fibroblast growth factor (bFGF) and VEGF promoting ingrowth of blood vessels. For a normal wound healing development, it is important to cease ongoing collagen production, its maximum deposition occurring around 21 days [22].

### The remodeling phase

The remodeling phase is the last phase of wound healing, it follows from approximately 3 weeks on after injury, and may last for 1 or 2 years. During this phase, the new tissues formed during the proliferative phase are remodeled to improve their integrity. Wound contraction and collagen remodeling characterize this phase. Fibroblasts are the central cell type in the remodeling phase and the hallmark of a successful remodeling is the conversion of collagen type III to type I. In order to achieve a normal healing, it is important to maintain a balance between synthesis and degradation [4].

The early deposition of collagen fibers is disorganized; however, over time the newly formed collagen matrix becomes further oriented and cross-linked. Fibroblasts upregulate the expression of the stronger type I collagen as remodeling occurs and simultaneously matrix metalloproteinase (MMPs) breakdown disordered old collagen, mainly of type III. A closely controlled balance between lysis and synthesis of collagen will result in the development of a normal scar in which the collagen fibers are rearranged in parallel bundles along tension lines, being the type I collagen predominant. This organization of the collagen matrix is accomplished through the last stages of the remodeling phase and this is due mainly to the wound contraction initiated in the proliferative phase. Fibroblasts increase the expression of α-smooth muscle actin and differentiate into myofibroblasts in response to cytokines such as TGF-β and to mechanical tension. Myofibroblasts will in turn contract the wound through the interaction of their integrin receptor with the ECM components such as fibronectin and collagen [19, 21].

The number of fibroblasts and macrophages is diminished by apoptosis as the wound heals. In addition, the growth of new capillaries stops and the metabolic activity at the wound microenvironment decreases. In the end of the wound healing process, a matured scar with high tensile strength is obtained [12].

## The macrophage in wound healing

### The macrophage

**Figure 2.**
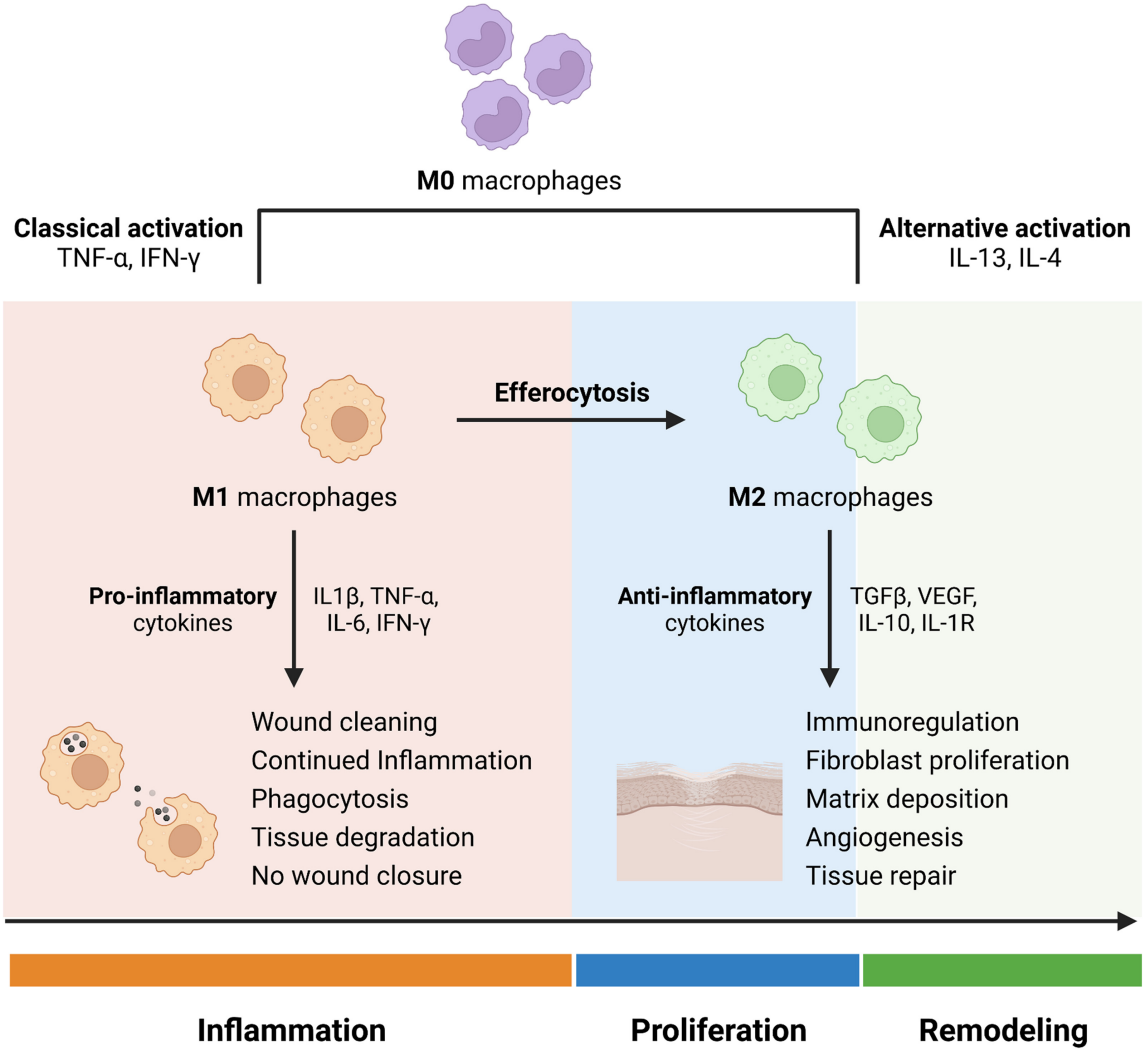
Macrophage polarization in wound healing. Macrophages can polarize to a M1 pro-inflammatory phenotype or to a M2 anti-inflammatory/reparative phenotype. In the early stages of wound healing, M1 macrophages predominate being activated by TNF-α or IFN-γ the local microenvironment. These macrophages are proficient phagocytes and remove wound debris. M1 macrophages clear apoptotic neutrophils through efferocytosis which is a key process in the macrophage phenotypic switch. M2 macrophages are stimulated by IL-13 or IL-4 and produce anti-inflammatory cytokines that act to switch off inflammation allowing the progression to the proliferation and remodeling phases. They stimulate fibroblast proliferation, matrix deposition and angiogenesis leading to tissue repair.

Macrophages have numerous roles during wound healing. Macrophages can polarize and acquire distinct phenotypes. The plasticity of these cells is crucial for wound repair [23]. Macrophages can polarize to M1 (pro-inflammatory), the classical activated form and to M2 (anti-inflammatory), the alternatively activated phenotype. The M1 macrophages act in pathogen phagocytosis and destroy/remove damaged cells, including neutrophils, whereas the M2 display repair and regeneration functions (Fig. 2). The M1 to M2 polarization, reflects the macrophage differentiation that causes a shift of the cells from inflammation to proliferation functions [24]. The macrophage phenotype changes as the wound heals and it is important to occur in an orderly manner for successful wound healing [11]. In reaction to different cues, macrophages can be classically activated—the M1 macrophages (stimulated by Toll-like receptors (TLR) ligands and interferon (IFN)-γ) or can be alternatively activated—the M2 macrophages (stimulated by IL-4/IL-13) [25, 26]. Macrophage polarization is influenced by the local cytokine milieu. Accordingly, a number of classes of macrophages have been described based on the production of specific factors, the expression of their surface markers, and on their biological activity [27–29]. The alternatively activated M2 macrophages are divided in three different subsets: M2a, M2b and M2c. M2a are induced by IL-4 or IL-13; M2b are prompted upon contact to immune complexes and agonists of TLRs or IL-1r and M2c by IL-10 and glucocorticoid hormones [28]. It is important to notice that the classification of macrophages into M1 and M2 subtypes represents an over-simplification, that a continuum of macrophage subtypes exists, and that these cells are able to revert back and forth according to the microenvironment [17].

According to distinct immune signals, different macrophage populations can be generated [17, 30, 31]:

- ‘Classically activated macrophages’ are effector macrophages that exhibit enhanced microbicidal capacity and have the capacity to secrete high levels of pro-inflammatory cytokines. These macrophages differentiate in response to TNF and IFN-γ. These macrophages have an important role in the host response, but their activation must be firmly controlled because their over-activation can lead to tissue damage caused by high levels of pro-inflammatory cytokines.
- ‘Wound-healing macrophages’ are differentiated in the presence of IL-4. They produce marginal amounts of pro-inflammatory cytokines and are less effective than classically activated macrophages at killing intracellular pathogens and producing reactive species. Importantly, for the wound healing process, these macrophages produce components of the ECM. A dysregulated activity of these macrophages can cause tissue fibrosis.
- ‘Regulatory macrophages’ have the key role of resolving immune responses and thus limit inflammation, due to the production of high levels of the immunosuppressive cytokine IL-10. The production of the regulatory cytokine TGF-β by macrophages following phagocytosis of apoptotic cells can lead to the generation of this type of macrophage.

### The role of the macrophage in wound healing

During the 1970s and the 1980s, several important studies were published regarding the important role of macrophages in the wound healing process. In addition and more recently, it also became clear that the depletion of macrophages would lead to an impaired healing process [32–34].

Macrophages are exclusive since they are involved in all phases of tissue repair and there is an evolution of their function during repair. At initial stages in the host response to tissue injury, macrophages react to the presence of pathogen-associated molecular patterns (PAMPs), DAMPs, and/or Th1 effectors and turn into classically activated M1 macrophages, which are characterized by the production of several pro-inflammatory mediators. Afterward, the microenvironment changes, due to the presence of IL-10, IL-13, and other mediators, and stimulates the alternative activation to M2 macrophages. These M2 macrophages release different factors, such as TGF-β1 and IL-10, that will promote immunosuppression and scar resolution. There must be a transition from pro-inflammatory to pro-reparative phenotype to allow the correct wound healing process. In the early host response to tissue damage, PMNs migrate to the wound area to clear debris and pathogens. Macrophages will then clear these apoptotic neutrophils in a process termed efferocytosis. This process is key for the macrophage phenotypic switch toward an M2 anti-inflammatory phenotype (Fig. 2) because macrophages that have engulfed neutrophils present lower levels of IL-12 and higher levels of IL-10 and TGF-β1 [35].

The macrophage phenotypic switch, from a pro-inflammatory to a pro-healing phenotype during the transition from the inflammatory to the proliferation phase, determines the wound healing progression and the outcome of the repair process [36]. It is described in the literature that about 85% of macrophages in the wounded tissue have an M1 pro-inflammatory phenotype in the early stages of repair, and a switch is observed by Days 5–7 to about 80–85% of anti-inflammatory M2 macrophages [37].

**Figure 3.**
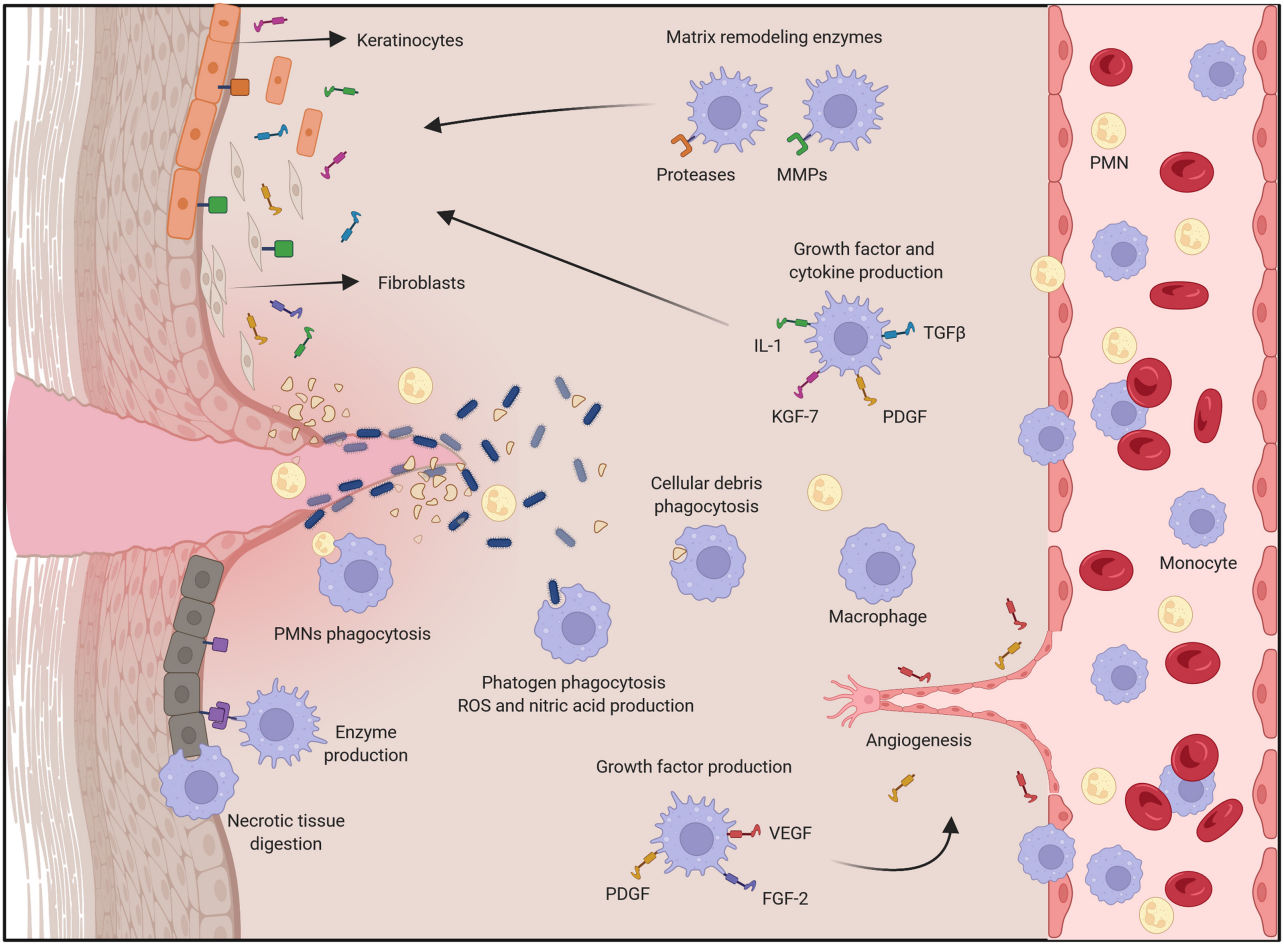
Macrophage roles in wound healing. Macrophages are key in the wound healing process since they play a central role in all stages of this process. They contribute to the wound cleaning and debridement and to the phagocytosis of pathogenic agents and they assist in the resolution of the inflammatory response through the phagocytosis of PMNs. They express growth factors that support angiogenesis. They also express cytokines and growth factors to induce cell proliferation, and deposition of extracellular matrix, as well as matrix remodeling enzymes.

Macrophages have many roles in the inflammatory, proliferative and remodeling phases of the wound healing process (Fig. 3). Macrophages are responsible for the: (i) Phagocytosis and destruction of pathogenic agents; (ii) Production of enzymes that will digest necrotic tissue; (iii) Phagocytosis and removal of cellular debris, dead cells, and necrotic tissue leading to the resolution of inflammation and shifting toward regeneration; (iv) Production of chemokines and growth factors such as for example PDGF, TGF-β and VEGF that will promote cell proliferation and new blood vessel development; (v) Promote the migration of endothelial cells and angiogenesis; (vi) Induce the recruitment of fibroblasts that will produce collagen and ECM, that will form the structural scaffold for the new tissue; (vii) Produce components of extracellular matrix; (viii) Synthesize matrix-remodeling enzymes [16, 38–41].

As key participants in the wound healing process, macrophages should be considered important therapeutic targets to advance wound healing. However, it is also important to take into consideration that disorders in macrophage function may lead either to delayed wound healing or to excessive wound healing in fibrosis [38, 39].

## What happens in chronic wounds?

Chronic wounds are characterized by a persistent inflammatory phase that prevents epidermal and dermal cells from reacting to chemical signals [42]. These wounds arrive to a state of pathologic inflammation due to a healing process that is incomplete or uncoordinated [13].

Chronic wounds do not go through the well-defined sequence of the healing phases: they are locked in the inflammatory phase that prevents the healing to progress to the proliferative phase (Fig. 1); and despite adequate wound management, they remain intractable. In these wounds, a hostile microenvironment is created and the correct balance among pro-inflammatory cytokines, chemokines, proteases and their inhibitors is disturbed [4].

The continuous inflammatory state of a chronic wound is characterized by profuse PMNs infiltration, together with its ROS and destructive enzymes that contribute to the prolongation of the inflammatory cycle [4]. This over-production of ROS leads to a direct damage of the ECM, cell membrane and consequently, premature cell senescence. Additionally, chronic wounds present high levels of Langerhans cells, pro-inflammatory macrophages and proteases, overexpression of inflammatory mediators and increased activity of matrix metalloproteinases, being all of these factors associated to clinical ulcer severity [42]. Activated macrophages and PMNs in the wounded area produce high levels of pro-inflammatory cytokines such as IL-1β and TNF-α that will cause an increase in MMPs production and at the same time reduce tissue inhibitors of MMPs (TIMPs). This imbalance will further augment the degradation of the ECM, will impair cell migration, and decrease fibroblast proliferation as well as collagen synthesis. The resulting products of the ECM breakdown will further promote inflammation, thus creating a perpetuated process [4].

Furthermore, chronic wounds became frequently more problematic due to the formation of bacterial biofilms, which additionally contribute to the perpetuation of the inflammatory phase [43].

There is strong evidence that macrophage dysfunction is an important factor in the pathogenesis of non-healing wounds [36]. For example, diabetic wounds present a dysregulated persistent pro-inflammatory M1 macrophage polarization while in normal wounds a transition to pro-healing M2 macrophages is observed about Day 3 after wounding [44, 45].

## Immunomodulatory biomaterials for wound healing

An immunomodulatory biomaterial can be defined as a material that is able to modulate immune responses and thus create a pro-regenerative microenvironment [46]. The concept behind biomaterial-based immunomodulation is to produce a biomaterial that facilitates the development of a microenvironment that will control the inflammatory response and promote tissue repair [47]. It is thus expected that immunomodulatory biomaterials will influence immune cell function and therefore stimulate tissue healing [48].

Macrophages, either tissue resident or monocyte-derived ones, are key in the wound healing process, therefore biomaterial-based strategies for wound healing are frequently focused on the macrophage and more specifically on macrophage polarization balance throughout the different phases of wound healing [11]. It is very important that a biomaterial used for wound healing applications does not promote a pro-inflammatory microenvironment. The biomaterial must stimulate the transition toward an M2-reparative macrophage phenotype. In the development of new wound healing therapies, biomaterials with immunomodulatory properties should be a key design factor [49].

**Figure 4.**
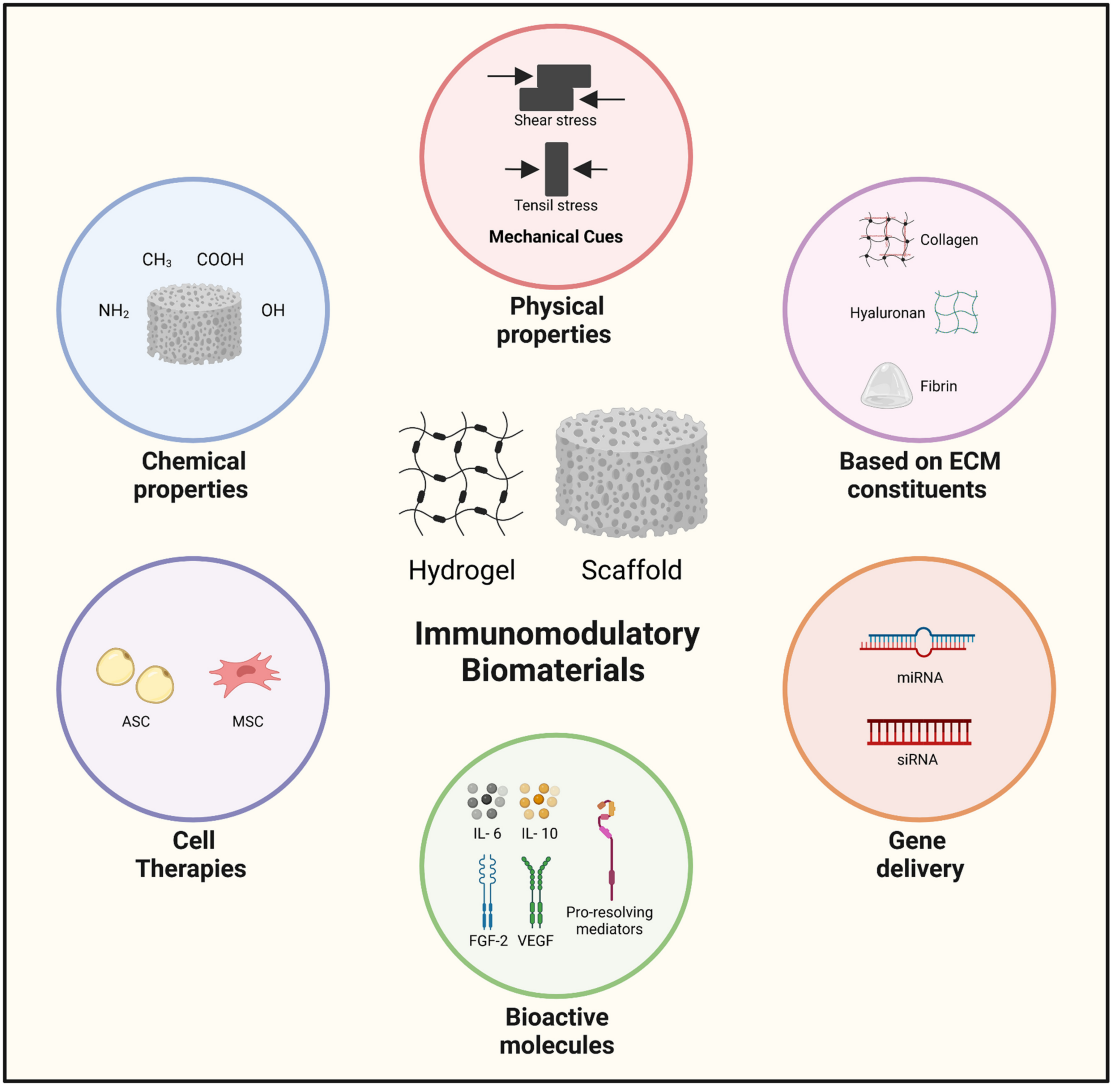
Strategies to develop immunomodulatory biomaterials. Several strategies can be used in the development of immunomodulatory biomaterials for wound healing applications: the modulation of their chemical properties; the tuning of the physical properties; the use of biomaterials based on extracellular matrix components; gene delivery; the incorporation of bioactive molecules; and cell therapies.

So far, it has not been proposed a clear classification for immunomodulatory biomaterials and, therefore, researchers of this field tend to describe and categorize these materials in terms of the different strategies that can be used for biomaterial-based immunomodulation. Briefly, some authors describe that immunomodulation can be achieved through chemical and physical modifications of the biomaterial and also through the delivery of bioactive molecules. Other authors provide more detailed categories for immunomodulation [47, 50, 51]. We have decided to group the biomaterials discussed in this work into the following categories to obtain immunomodulatory effects: (i) Changing the physical or chemical properties of the material; (ii) Developing biomaterials based on decellularized ECM; (iii) Incorporating bioactive molecules such as, for example, anti-inflammatory drugs or growth factors; (iv) Using cell therapy methods and (v) gene delivery (Fig. 4).

We have performed a thorough literature review on the development of immunomodulatory biomaterials for wound healing applications and we have selected some recent and interesting examples that are summarized in Table 1. We have decided to include only examples of biomaterials that revealed to have immunomodulation capacity when tested in an *in vivo* model of wound healing.

**Table 1.** Immunomodulatory biomaterials for wound healing applications

Immunomodulatory biomaterial	Cargo	Outcome	Reference
Collagen type I and sulfated chitosan hydrogel	–	Facilitated polarization of M1-to-M2 macrophages Equilibrated pro-inflammatory and anti-inflammatory cytokines.	[57]
High-sulfated hyaluronan and collagen hydrogel	–	Increased pro-regenerative macrophage activation	[58]
Sponge of low-degree-sulfated κ/β-carrageenan	–	Secretion of anti-inflammatory factors Induced macrophage toward M2	[59]
Silk fibroin hydrogel	–	Expression of TNF-α and CD163 genes Deposition and remodeling of collagen type I and III fibers	[60]
Silk fibroin hydrogel + silica microspheres + silver nanoparticles	–	Transition from M1 to M2 macrophage phenotype Inhibition of the formation of neutrophil extracellular traps Decrease in of pro-inflammatory factors	[61]
Dextran-based hydrogel + black phosphorus nanosheets + zinc oxide nanoparticles.	–	Macrophage polarization toward M2 phenotype Secretion of anti-inflammatory cytokines	[62]
Elastin/Gelatin hydrogel	–	Predominance of M2 macrophages Increased angiogenesis and collagen deposition	[63]
Hybrid hydrogel of glycyrrhizic acid and silk fibroin + Zn^2+^	–	Decreased neutrophils infiltration Decreased expression of pro-inflammatory cytokines	[64]
Placenta-derived decellularized ECM hydrogel	–	Upregulation of anti-inflammatory cytokines Downregulation of pro-inflammatory cytokines	[65]
Gelatin methacryloyl hydrogel	IL-6	Increased survival of skin allografts	[66]
Po(lylactic acid) electrospun fibers	IL-10	Macrophage polarization toward a M2 phenotype	[67]
Hyaluronic acid	anti-TNF-α	Decreased macrophage infiltration Decreased IL-1β levels on Day 1	[68]
Alginate hydrogel	FGF-2	Shift toward M2 phenotype Changes in the cytokine profile	[69]
Chitosan–silver hydrogel	bFGF	Stimulated collagen deposition Promoted M2 macrophage polarization	[70]
Hyaluronic acid, dextran and β-cyclodextrin hydrogel	Resveratrol VEGF plasmid	Decreased levels of IL-1β and TNF-α gene expression.	[71]
Gelatin-oxidized starch nanofibers	*Lawsonia Inermis L.* extracts	Reduced inflammatory response. Decreased levels of pro-inflammatory macrophages	[72]
Hyaluronic acid hydrogel	Extracellular vesicles from mesenchymal stem cells	Promoted M2 macrophage polarization	[73]
Poly(urethane acrylate) patch	Human dermal fibroblast	Increased secretion of angiogenic factors Increased activity of inflammatory cytokines for M2 macrophages polarization	[74]
Chitosan–polyurethane hydrogel	Adipose-derived adult stem cells	Increased ratio of M2/M1. Increased production of TGFβ-1 and SDF1 Decreased production of TNF-α and IL-1β.	[55]
Fibrin hydrogel	Silver sulfadiazine Adipose-derived stem cells	Reduced expression of TNF-α. Increased neovascularization Increased collagen deposition	[75]
*Aloe Vera* hydrogel	Adipose-derived stem cells	Decreased levels of TGF-β1 and IL-1β (Day 7) High levels of TGF-β1 in the treated wounds (Day 14)	[76]
Collagen scaffold	(miR)-29B	Improved collagen type III/I ratio Higher matrix metalloproteinase (MMP)-8/tissue inhibitor of metalloproteinase (TIMP)-1	[78]
β-cyclodextrin and poly(amidoamine) polymer	MMP-9siRNA	Increased content of collagen around the wounded tissues. Decreased infiltration of PMNs leukocytes	[79]

Even though we have made an extensive literature analysis including all types of biomaterials, it is important to notice that the herein presented immunomodulatory biomaterials are almost all hydrogels. This is due to the fact that hydrogels have interesting and adequate properties for wound healing applications.

Hydrogels are 3D hydrophilic interconnected polymeric networks that can be either of natural or synthetic origin, and have the ability to incorporate high amounts of water. Hydrogels have the capability to create a microenvironment that resembles the ECM, therefore making them quite interesting for tissue repair and regeneration applications [52, 53]. Hydrogels, exhibit unique features for wound healing applications since they mimic the skin microenvironment, due to their porous and hydrated structure. Additionally, they also contribute to the formation of a physical barrier against pathogenic agents, and to some extent remove excess exudate. Hydrogels provide a moisture environment that promotes the healing process, and can perfectly fill irregularly shaped wounds [54, 55]. Moreover, hydrogels can also be used as delivery systems for the sustained release of bioactive molecules, cells and genes [3]. However, since hydrogels have a high water content, they have a poor exudate absorptive capacity. Furthermore, they are also difficult to handle due to low mechanical properties [56].

Taking into consideration the ‘tuning of the chemical or physical properties’ of a biomaterial or the use of ‘biomaterials based on ECM constituents’ to acquire immunomodulatory properties, Shen *et al.* [57] developed a hydrogel based on collagen type I and sulfated chitosan, which had a positive effect in the resolution of chronic inflammation in diabetic wounds, and thus accelerated diabetic wound healing. The developed hydrogel facilitated the polarization of M1-to-M2 macrophages and equilibrated the content of pro-inflammatory and anti-inflammatory cytokines. Hauck *et al.* [58] developed an immunomodulatory hyaluronan/collagen-based hydrogel containing high-sulfated hyaluronan to act as immunoregulatory component to modulate the inflammatory macrophages in disturbed wound healing. The hydrogels were effective in reducing inflammation, and in increasing pro-regenerative macrophage activation, as well as in accelerating new tissue formation and wound closure. Song *et al.* [59] prepared a sponge dressing for wound healing using low-degree-sulfated κ/β-carrageenan oligosaccharide and evaluated its immunomodulatory effects. This sponge dressing promoted the secretion of anti-inflammatory factors and induced the polarization of lipopolysaccharide (LPS)-activated macrophages toward M2 type. Acceleration of the healing process of skin wounds in diabetic rats was also observed. Chouhan *et al.* [60] developed an *in situ* forming hydrogel of silk fibroin for the treatment of burn wounds. They have observed that their hydrogel promoted the transition from the inflammatory to the proliferative stage through the expression of TNF-α and CD163 genes. They also observed deposition and remodeling of collagen type I and III fibers indicating an enhanced tissue repair. Mei *et al.* [61] produced a silk fibroin hydrogel system, co-encapsulated with metformin-loaded mesoporous silica microspheres and silver nanoparticles. When applied in a wounded diabetic mouse model, a shift from M1 to M2 macrophage phenotype was observed. In addition, an inhibition of the formation of neutrophil extracellular traps and a decrease in the release of pro-inflammatory factors from neutrophils was observed. Fibroblast migration and angiogenesis were enhanced. Zhou *et al.* [62] reported a dextran-based hydrogel composed of methacrylated gelatin and dextran loaded with black phosphorus nanosheets and zinc oxide nanoparticles. When the hydrogels were applied in an *in vivo* model of wound healing, the authors observed macrophage polarization toward a M2 phenotype and secretion of anti-inflammatory cytokines. Tian *et al*. [63] designed an *in situ* formed elastin/gelatin hydrogel and in a mice wound model observed a predominance of M2 macrophages, and increased angiogenesis and collagen deposition, making this hydrogel interesting for wound repair. Qian *et al.* [64] developed a new hybrid hydrogel composed of glycyrrhizic acid, silk fibroin and inorganic Zn^2+^. This hybrid hydrogel was tested in a diabetic rat wound model and it was observed a substantial decrease in the infiltration of neutrophils and in the expression of pro-inflammatory cytokines in the wound area. Wang *et al*. [65] explored the use of glycosaminoglycans for wound healing applications and developed a hydrogel from placenta-derived decellularized ECM. The hydrogel revealed to have anti-inflammatory effects inducing an upregulation of anti-inflammatory cytokines together with a downregulation of pro-inflammatory cytokines in a skin wound healing model.

Regarding the ‘incorporation of bioactive molecules’ to develop new materials with immunomodulatory properties, there is a very large number of bioactive molecules with promising properties to be explored in wound healing applications. The majority of the studies reported the use of anti-inflammatory cytokines or the growth factors VEGF and FGF. Uehara *et al.* [66] devised a gelatin methacryloyl IL-6 eluting hydrogel to be applied in the interface between the wound and the skin allograft. The gradual elution of IL-6 lead to a reduction in the inflammatory reaction around the allograft and consequently these skin allografts presented a more favorable outcome. Chen *et al.* [67] establish an IL-10-loaded electrospun poly(lactic acid) fibers with cascade release behavior. The initial release of IL-10 prevented an excessive inflammatory response whereas the subsequent release maintained high levels of IL-10 in the wound to allow macrophage polarization toward a M2 phenotype. Friedrich *et al.* [68] developed an interesting approach using monoclonal antibodies: they tested a topical application of anti-TNF-α with hyaluronic acid in a rat burn model. They observed a decrease in macrophage infiltration together with a decrease in IL-1β levels on Day 1 post-injury. Das *et al.* [69] described an alginate hydrogel that delivers syndecan-4 proteoliposomes (‘syndesomes’) with fibroblast growth factor-2 (FGF-2) for enhanced wound healing. This hydrogel had an immunomodulatory effect on wound macrophages, leading to a shift toward the M2 phenotype, together with changes in the cytokine profile. Xuan *et al.* [70] developed a chitosan–silver hydrogel with basic fibroblast growth incorporated for the treatment of infected wounds. Besides being effective in bacterial inhibition, this hydrogel promoted collagen deposition and promoted M2 macrophage polarization leading to a reduction of the inflammatory response. Wang *et al.* [71] reported the fabrication of a hydrogel using hyaluronic acid, dextran and β-cyclodextrin loaded with resveratrol and vascular endothelial growth factor plasmid. This hydrogel, when applied in wound, inhibits the inflammatory response exhibiting lower levels of IL-1β and TNF-α gene expression. Hadisi *et al.* [72] produced gelatin-oxidized starch nanofibers containing *Lawsonia Inermis L.* (also known as henna) herbal extracts, with the aim of treating second-degree burn wounds. Among other observations, the immunohistochemical studies revealed that burn wounds treated with these nanofibers presented a reduced inflammatory response and a decrease in the numbers of pro-inflammatory macrophages. Yang *et al.* [73] created a hyaluronic acid hydrogel with extracellular vesicles derived from mesenchymal stem cells. When applied in the wound of a mouse skin injury model, it drives macrophages toward an M2 anti-inflammatory phenotype.

The use of ‘cell therapies’ combined with biomaterials to achieve immunomodulation has also been reported. Kim *et al.* [74] introduced a biocompatible poly(urethane acrylate) flat patch with specifically designed holes where spheroids of human dermal fibroblast were incorporated. This strategy lead to an increased secretion of angiogenic factors together with an increased activity of inflammatory cytokines for M2 polarization of macrophages. Adipose stem cells (ASCs) are considered to increase wound healing ability since they have the potential to secrete growth factors and to differentiate into various cell lineages, therefore several hydrogels with ASCs have been developed. Chen *et al.* [55] demonstrate that chitosan–polyurethane hydrogel and cryogel containing ASCs promoted wound healing in a diabetic skin wound model. The application of these biomaterials increased the ratio of M2/M1 macrophages, together with an enhanced production of TGFβ-1 and stromal cell-derived factor 1 (SDF1) and a decrease in the pro-inflammatory cytokines TNF-α and IL-1β. Banerjee *et al*. [75] developed a sequential treatment for burn wounds consisting of an initial application of a fibrin hydrogel containing silver sulfadiazine loaded chitosan microsphere, followed by the application 9 days later of a fibrin hydrogel with ASCs. This sequential treatment significantly reduced the expression of the pro-inflammatory cytokine TNF-α, increased neovascularization markers and dermal collagen matrix deposition. Oryan *et al*. [76] evaluated *in vivo* the effects of an *Aloe vera* hydrogel loaded with ASCs on a rat burn wound model. They observed a decrease in the TGF-β1 and IL-1β at Day 7, together with high levels of TGF-β1 at Day 14 in the treated wounds.

The use of gene delivery to engineer the desired immune response is increasing. It can be used for the direct delivery of factors that have immunomodulatory properties in the wounded area. Recently, the delivery of small interfering RNA (siRNA) and microRNA (miRNA) is being explored with the aim of decreasing the expression of a target gene. They cause gene silencing effects at the post-transcriptional level by directing mRNA. Both siRNA and microRNA are small molecules. siRNAs are highly specific having an unique mRNA target, whereas microRNAs have several targets making them very interesting for the treatment of various diseases and also for wound healing applications [77]. Concerning immunomodulation based on ‘gene delivery’, Monaghan *et al.* [78] developed a collagen scaffold loaded with miRNA-29B with the aim of modulating the ECM after cutaneous injury. These scaffolds reduced collagen type I production and improved collagen type III/I ratios and a significantly higher matrix metalloproteinase (MMP)-8: tissue inhibitor of metalloproteinase (TIMP)-1, suggesting increased matrix turnover and showing the potential benefit of combining exogenous miRs with collagen scaffolds to improve extracellular matrix remodeling following injury. Li *et al*. [79] presented a strategy to decrease MMP-9 expression and improve diabetic wound through RNA interference, for that a star-branched cationic polymer with β-cyclodextrin and poly(amidoamine) to carry siRNA to interfere with MMP-9 expression was developed. They were able to increase the content of collagen around the wounded tissues, as well as decrease the infiltration of PMNs leukocytes. Wounded tissues are in an inflammatory state, which is associated with increased infiltrating macrophages that are a source of MMPs. MMPs are enzymes that have the ability to selectively degrade components of the ECM. For example, MMP-2 and MMP-9 degrade type IV collagen and MMP-8 has the capability to degrade collagens type I, II and III and is the predominant collagenase in healing wounds. In the context of wound healing; MMPs and their inhibitors, TIMPs, play important roles in the degradation and regeneration of wounded tissues. The correct balance between MMPs and TIMP is crucial for an effective tissue repair [80]. On the other hand, an imbalance of MMPs and TIMP leads to an increased ECM degradation together with decreased fibroblast proliferation and collagen synthesis [4].

As discussed above, several different strategies to achieve biomaterials with immunomodulatory capacity revealed to be effective in terms of modulating the inflammatory response, mainly through macrophage polarization toward an anti-inflammatory phenotype and through the increase of the levels of anti-inflammatory cytokines together with decreased levels of pro-inflammatory cytokines. There are no clear evidences that a particular strategy for immunomodulation produced better results than another. The herein discussed immunomodulatory biomaterials revealed to have at some extent the ability of creating pro-regenerative microenvironments and have a positive effect on the wound healing process, stressing the relevance of immunomodulation in tissue repair and regeneration.

The process of wound healing is complex, therefore it is likely that a single therapeutic target will not be enough. This highlights the necessity of develop new therapies with a multidimensional approach able to target the different phases of wound healing [19]. We hypothesize that by addressing sequentially the different phases of wound healing and, therefore, target the different cell types involved in this process, a better therapeutic outcome will be achieved than those currently available.

## Conclusions and future perspectives

The problem of chronic wounds seriously threatens the quality of life of millions of people around the world. Non-healing chronic wounds present an accumulation of M1 pro-inflammatory macrophages owing to an ineffectiveness to overcome the inflammatory phase and advance to the proliferative and remodeling phases. For that reason, biomaterials that are able to modulate macrophage polarization and induce a shift from M1 to M2 phenotype are currently considered as ideal candidates to accelerate wound healing [43].

This review has recalled recently developed immunomodulatory biomaterials to be used to enhance wound healing. The findings herein described have demonstrated the clear potential of directing the immune system to improve chronic wound healing.

Biomaterials developed for wound dressings are capable of incorporating several different molecules that have a role in the wound healing process such as cytokines, antibodies, growth factors and anti-inflammatory molecules among others. The encapsulation of cells within these biomaterials is also possible as well as gene delivery [10, 56]. Therefore, there are a myriad of possible biological targets to be explored and thus exciting new developments in this area of research are expected at any time.

Clearly, a thorough evaluation on the *in vivo* wound microenvironment throughout the different phases of healing, a better understanding on the contribution of the spatiotemporal macrophage polarization profile as well as their functional contribution during all process, and a detailed evaluation of the herein described biomaterials in terms of their immunomodulatory effects both at the inflammatory response and at the tissue healing process would represent a significant advance in the fight against chronic wounds.
